# Papillary Thyroid Carcinoma Variants are Characterized by Co-dysregulation of Immune and Cancer Associated Genes

**DOI:** 10.3390/cancers11081179

**Published:** 2019-08-15

**Authors:** Jaideep Chakladar, Wei Tse Li, Michael Bouvet, Eric Y. Chang, Jessica Wang-Rodriguez, Weg M. Ongkeko

**Affiliations:** 1 Otolaryngology-Head and Neck Surgery, Department of Surgery, University of California, San Diego, La Jolla, CA 92093, USA; 2 Department of Surgery, University of California, San Diego, La Jolla, CA 92093, USA; 3 Moores Cancer Center, University of California, San Diego, La Jolla, CA 92093, USA; 4 Surgery Service, VA San Diego Healthcare System, San Diego, CA 92161, USA; 5 Department of Radiology, University of California, San Diego, California CA 92093, USA; 6 Radiology Service, VA San Diego Healthcare System, San Diego, CA 92161, USA; 7 Department of Pathology, University of California, San Diego, La Jolla, CA 92093, USA; 8 Pathology Service, VA San Diego Healthcare System, San Diego, CA 92161, USA

**Keywords:** papillary thyroid carcinoma, cancer immunology, microRNA

## Abstract

Papillary thyroid carcinoma (PTC) variants exhibit different prognosis, but critical characteristics of PTC variants that contribute to differences in pathogenesis are not well-known. This study aims to characterize dysregulated immune-associated and cancer-associated genes in three PTC subtypes to explore how the interplay between cancer and immune processes causes differential prognosis. RNA-sequencing data from The Cancer Genome Atlas (TCGA) were used to identify dysregulated genes in each variant. The dysregulation profiles of the subtypes were compared using functional pathways clustering and correlations to relevant clinical variables, genomic alterations, and microRNA regulation. We discovered that the dysregulation profiles of classical PTC (CPTC) and the tall cell variant (TCPTC) are similar and are distinct from that of the follicular variant (FVPTC). However, unique cancer or immune-associated genes are associated with clinical variables for each subtype. Cancer-related genes *MUC1*, *FN1*, and *S100*-family members were the most clinically relevant in CPTC, while *APLN* and *IL16*, both immune-related, were clinically relevant in FVPTC. *RAET*-family members, also immune-related, were clinically relevant in TCPTC. Collectively, our data suggest that dysregulation of both cancer and immune associated genes defines the gene expression landscapes of PTC variants, but different cancer or immune related genes may drive the phenotype of each variant.

## 1. Introduction

Thyroid carcinoma is currently the fastest growing cancer in the world and is the most common endocrine carcinoma in the Western world [1]. The yearly occurrence of thyroid cancer has more than doubled in the past two decades, with most of this growth being attributed to papillary thyroid carcinoma (PTC), which constitutes over 80% of all thyroid cancer cases [1]. Papillary thyroid carcinoma cases are divided into subtypes differentiated by physiology and clinical outcomes. The three most common subtypes consist of classical (CPTC), follicular-variant (FVPTC), and tall cell (TCPTC) PTC, with CPTC being the most common subtype and TCPTC being the least common subtype. Clinical outcomes vary between these subtypes. Most notably, TCPTC is characterized by higher rates of mortality, tumor recurrence, distant metastasis, and lymph node metastasis than the other subtypes [2]. FVPTC is much more benign, as it has been correlated with much lower mortality and metastasis rates than CPTC and TCPTC [2]. A recent study of FVPTC has resulted in further sub-classification of this phenotype into the invasive (iFVPTC) and noninvasive (niFVPTC), also called encapsulated, sub-variants [3]. niFVPTC has been reclassified as noninvasive follicular thyroid neoplasm with papillary-like nuclear features (NIFTP) because it resembles benign tumors more than carcinomas [4,5]. However, this classification is largely based on pathological tumor features, with little known about the genotypic differences between the sub-variants.

Despite increases in thyroid cancer cases, few mechanisms that differentiate between PTC subtype pathogenesis and prognosis have been identified. The most prominent mechanism studied is the point mutation of the *BRAF* proto-oncogene, which results in the expression of the BRAF-V600E mutant protein. This protein promotes tumorigenesis by activating the mitogen-activated protein kinase (MAPK) signaling pathway [6]—a pathway crucial for cell proliferation and survival. This mutation has been shown to be present predominantly in CPTC and TCPTC patients and is correlated with an aggressive phenotype contrary to the slow-growing nature of PTC in general. Additionally, the mutant *BRAF* protein may have synergistic effects with other mutations, namely through the newly identified relationship it has with telomerase reverse transcriptase (*TERT*) promoter mutations [7]. It has been demonstrated that clinical prognosis is significantly worse when the *BRAF* mutation is present along with a TERT mutation, complicating the role that *BRAF* has in thyroid cancer [7].

The second most prevalent mutation is on *RAS* and its isoforms, *HRAS, NRAS*, and *KRAS*. These mutations are dual activators of the MAPK and the phosphatidylinositol 3-kinase/Akt (PI3K/AKT) pathways, but more commonly activate the latter pathway in promoting tumorigenesis in FVPTC patients [8].

Treatment of thyroid cancer has high success rates, with relatively low tumor recurrence. Currently, the most common treatments include surgery and radioactive iodine therapy, and to a lesser extent, chemotherapy, external beam radiation, and targeted therapy [9]. However, the aforementioned treatments come with their own problems despite their efficacy. Thyroidectomy is limited to non-metastatic tumors and carries with it the inherent risks of surgery. Radioactive iodine therapy, although effective for most non-invasive tumors, has varying effectiveness when applied to PTC, as thyroid tumors are able to develop a resistance to the uptake of iodine. This is caused by the mutant BRAF-V600E protein, which mediates the silencing of the sodium/iodine symporter via the up-regulation of certain epigenetic pathways [10], thereby rendering radioactive iodine ineffective from a treatment standpoint. Although chemotherapy is the most widespread and effective option for eradicating late-stage carcinomas, its accompanying morbidity is well documented. The current most prevalent targeted therapy approved by the FDA for thyroid cancer is the administration of the BRAF inhibitor dabrafenib combined with the MAPK inhibitor trametinib [11]. Although promising in theory, this combination is only effective for carcinomas that test positive for the BRAF mutation, which is not necessarily present in all CPTC or TCPTC tumors. Furthermore, in studies conducted on thyroid cancer patients who had the *BRAF* mutation, it was found that only 4% of patients experienced a complete response when exposed to this new therapy regimen [12].

Because of the drawbacks and varying patient responses to current cancer modalities, the application of immunotherapy may also be a promising alternative option. Potential immunotherapies have revolved around exploiting checkpoint inhibitors involved in immune pathways associated with cancer progression, best exemplified by recent findings identifying the usage of checkpoint inhibitors and CAR-T as an effective way to increase drug effectiveness in patients [13,14]. In previous studies by our lab, thyroid cancer was identified as one of the most immunogenic cancers, suggesting potential for effective immune therapies in PTC treatment (Li et al, paper submitted for publication). However, there are few conclusive studies that identify novel targets for immunotherapy within the genome and transcriptome.

In this study, we analyzed the co-dysregulation of immune-associated (IA) and cancer-associated (CA) genes in the different subtypes of PTC using a multi-scale approach, using data provided by The Cancer Genome Atlas (TCGA). We first identified gene dysregulation on the scale of RNA expression. We then examined these dysregulations for significance on the scales of clinical variables correlations, copy number variation/aneuploidy correlations, mutation correlations, and micro-RNA regulation to identify key IA and CA genes that differentiate PTC subtypes from each other. In addition, on a pathways scale, we clustered IA and CA genes based on function to determine coordinated dysregulations of biological processes that define each PTC subtype.

## 2. Results

### 2.1. Similarities and differences between gene dysregulation landscapes of PTC subtypes

**Figure 1:**
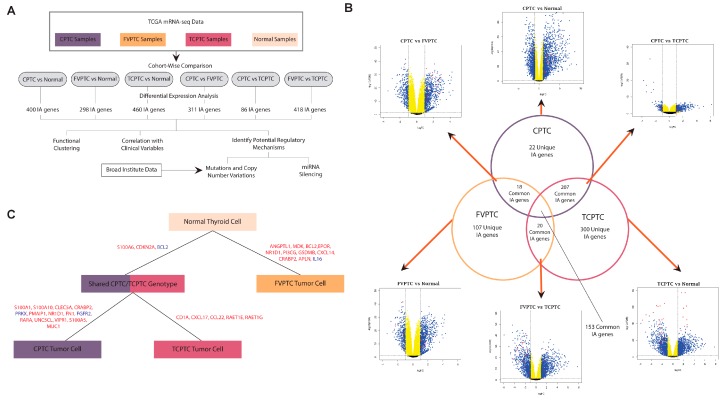
Study overview and differential expression analysis. (**A**) Schematic of analyses and workflow. (**B**) Venn diagram illustrating the unique and common significantly dysregulated IA genes after differential expression analysis of samples from each type vs. normal samples. Volcano plots depict statistical significance and fold change of differential expression analyses for all CA and IA genes. Red datapoints represent genes with expression that correlates with MACIS score. (**C**) Schematic of dysregulated genes that characterize each PTC subtype. Both differential expression results and clinical variable correlations results were used to determine the most important genes to each subtype. Gene names labeled in blue represent downregulation in a subtype and red represents upregulation in a subtype.

RNA-sequencing data from The Cancer Genome Atlas (TCGA) for four sample cohorts, one of each PTC subtype and one of solid tissue normal samples, were downloaded. Sequencing data were used to identify significantly dysregulated genes between groups in each cohort by utilizing differential expression analysis (FDR <0.05, Figure 1A, Tables S1–S6). The genes were then filtered using a list of known IA and CA genes, so only genes implicated in immune or cancer-related processes were retained as candidates for further analyses. The lists of dysregulated genes from each cohort were compared to identify similarities and differences between cohorts (Figure 1B).

A total of 153 genes were significantly dysregulated in all three subtypes when compared to normal samples (Figure 1B). 207 genes were dysregulated in both CPTC and TCPTC, 18 genes were dysregulated in both FVPTC and CPTC, and 20 genes are dysregulated in both FVPTC and TCPTC. This suggests that the landscape of IA and CA dysregulation is most similar between TCPTC and CPTC. Interestingly, 300 genes were dysregulated solely in TCPTC, while only 22 were dysregulated solely in CPTC, suggesting that while TCPTC is most similar to CPTC in gene dysregulation, it is still relatively distinct compared to the other subtypes. A larger number of genes are dysregulated in TCPTC than in other subtypes. 

From volcano plots of differential expression results, we observe that the fold changes of most dysregulated genes in FVPTC were not significant (|logFC| > 1), whereas the fold changes of IA and CA genes are much higher in the other two subtypes (Figure 1B). These results suggest that FVPTC and normal tissue are relatively similar, and the lack of high fold-change dysregulation may explain the relatively benign phenotype of FVPTC. 

Due to the important differences between the newly classified variants of FVPTC, we attempted to investigate immune-associated elements that may differ between them. TCGA data was limited in this regard as FVPTC patients were not further classified into subtypes. We looked to sequencing data from a previous study, Pool et al. [15], and performed differential expression analysis on niFVPTC vs iFVPTC samples (Figure S1). The genotypes of both sub-variants were similar, as evidenced by the small number of differentially expressed genes. Only a limited number of IA genes were found to be significantly dysregulated between the two sub-variants. We therefore found that the determining factor of FVPTC morphology may not be related to immune-associated elements. 

Using differential expression data, clinical variable correlations, genomic alteration correlations, and pathways associations, we have compiled a list of genes most implicated in each PTC subtype and that critically define the molecular difference between the subtypes (Figure 1C). Our most salient findings will be presented in the sections below.

### 2.2. Filtering differentially expressed genes by MACIS score

Due to the low patient mortality rate that is characteristic of thyroid cancer in general, differentially expressed genes were further filtered by patient prognosis using the MACIS scoring system. After MACIS scores were divided into four categories, the Kruskal-Wallis test was used to analyze a trend in potential patient mortality that corresponds with a direction of gene dysregulation (*p* < 0.05). Genes exhibiting a significant correlation were denoted on the volcano plots (Figure 1B). We note that most of the genes with high statistical significance of differential expression (y-axis on volcano plots) exhibited correlation with MACIS score in the TCPTC vs. normal tissue comparison, which may indicate their ability to confer the more aggressive phenotype of TCPTC. Only MACIS-score correlated genes were candidates for further analysis.

### 2.3. MUC1, FN1, S100 family genes, and CA/IA pathways define the CPTC dysregulation landscape

**Figure 2:**
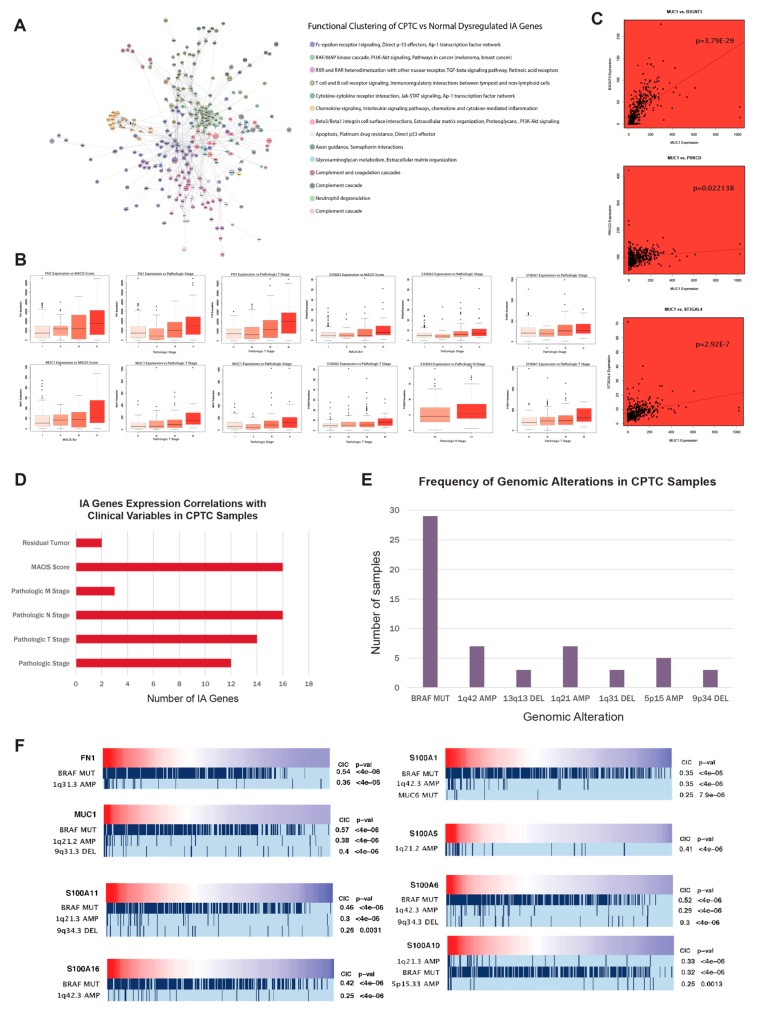
Pathways and genes characterizing the CPTC dysregulation landscape. (**A**) ReactomeFIViz functional clustering analysis for dysregulated genes in CPTC vs normal samples. CA or IA gene nodes are distinguished by black text and red or blue outlines, with red signifying gene upregulation and blue signifying gene downregulation in CPTC. Node size correlates with larger absolute logFC. Edges of the plots were determined by Functional Interaction (FI) score visualization. (**B**) Boxplots presenting association of dysregulated genes’ expression with clinical variables for CPTC patients. (**C**) Scatterplots correlating expression of MUC1 to that of related genes. (**D**) Barplot documenting number of CA/IA genes that correlated with six clinical variables. (**E**) Barplot depicting number of samples that possess the most common genomic alterations. (**F**) REVEALER plots illustrating genomic alteration associated with dysregulated genes. The REVEALER analysis was conducted using CPTC samples only.

The Cytoscape ReactomeFIViz plugin [16] allowed us to create functional clusters of dysregulated genes, with the purpose of grouping genes based on similarity of function in relation to biological pathways. Both CA and IA pathways are strongly implicated in the dysregulation landscape of CPTC, with the largest cluster of genes being linked to both the IA pathway of Fc-epsilon receptor signaling and the CA pathways of p53 effectors and Ap-1 transcription factor network (Figure 2A). CA pathways of PI3K-Akt signaling, MAP kinase cascade, TGF-beta signaling, and retinoic acid signaling account for the next two largest functional clusters, while IA pathways of lymphocyte signaling and cytokine signaling account for the subsequent three largest functional clusters.

Expressions of CA genes *MUC1*, *FN1*, and genes in the *S100*-family are highly correlated with pathologic stage and pathologic TNM stages of CPTC patients (Figure 2B). While these genes have been implicated in PTC [10,17,18], no significant correlation with clinical variables was observed in other PTC subtypes.

We next correlated the expressions of dysregulated genes to other genes in the same pathway to investigate possible co-expression (Spearman correlation test, *p* < 0.05). Notably, *MUC1* exhibited co-upregulation relationships with the IA genes *B3GNT3* and *PRKCD*, underscoring the importance of interaction between CA and IA pathways in CPTC (Figure 2C). *MUC1* expression is also correlated with expression of *ST3GAL4*. Both *B3GNT3* and *ST3GAL4* play a vital role in the mediation of glycosylation, which has been linked to drug resistance and cancer progression of a host of epithelial cancers [19]. 

Finally, we discovered a significant number of correlations of IA gene expression to six clinical variables in CPTC, greater than the number of correlations observed in the other two subtypes (Figure 2D).

### 2.4. MUC1, FN1, and S100 family genes in CPTC correlated to alterations at tumor-associated loci

To identify copy number variations (CNVs) and mutations that are commonly associated with the dysregulation of IA genes, the REVEALER algorithm was used [20], utilizing BROAD Firehose genomic alteration files and gene expression data. CNV or mutation presence was considered to be significantly associated with gene dysregulation if the CIC values output by the algorithm were greater than 0.25 [20]. 

In CPTC, a majority of the genes analyzed showed a correlation between direction of dysregulation and *BRAF* mutation presence (Figure 2F). Apart from the *BRAF* mutation, the most common CNVs associated with gene dysregulation were located at the 1q21 locus, which has been previously identified to be associated with cancer progression [21]. Amplifications at the 1q42 and 5p15 loci and deletions at the 9q34 locus were also commonly associated with gene dysregulation. Studies on the 1q42 locus have associated it with the progression of hereditary prostate cancer [22] and melanoma [23]. Lung adenocarcinoma susceptibility has been mapped to the 5p15 locus [24]. Deletions in at the 9q34 locus are heavily implicated in causing sporadic bladder cancer [25]. Of importance may be the commonalities between alterations correlated to genes in the *S100*-family. These genes are commonly correlated with 1q21 and 1q42 amplifications, which are also the most frequent genomic alterations in CPTC aside from *BRAF* mutation (Figure 2E). Interestingly, no occurrences of the *BRAF* mutation occurred alongside a *TERT* promoter mutation, suggesting a relative rarity of this mutation in comparison to its frequency in more aggressive thyroid cancers.

### 2.5. APLN, IL16, and CA/IA pathways define the FVPTC dysregulation landscape

**Figure 3:**
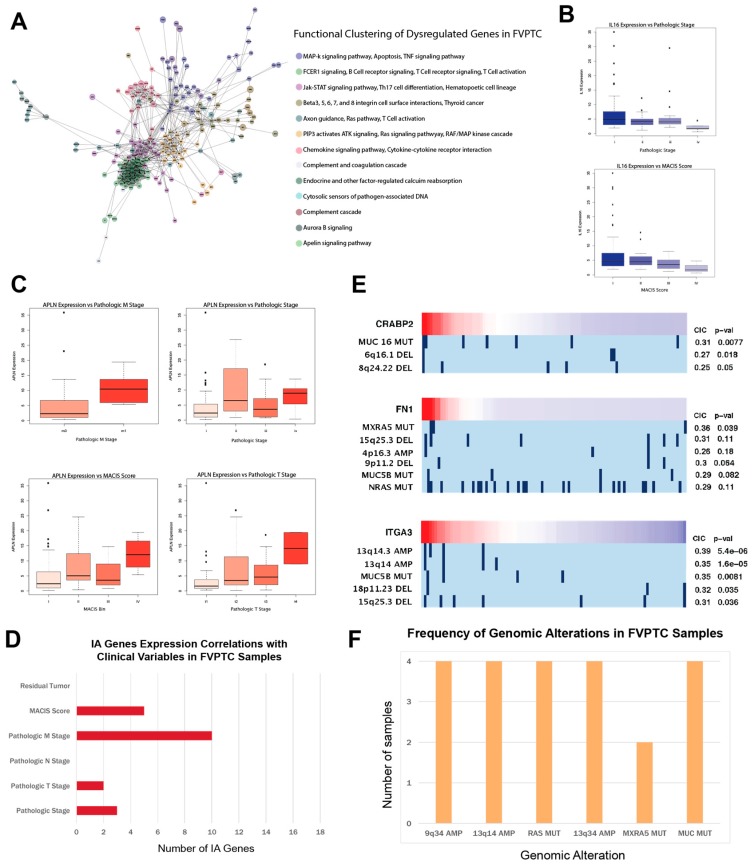
Pathways and genes characterizing the FVPTC dysregulation landscape. (**A**) ReactomeFIViz functional clustering analysis for dysregulated genes in FVPTC vs normal samples. (**B**) Boxplots presenting association of *IL16* expression with clinical variables for FVPTC patients. (**C**) Boxplots presenting association of *APLN* expression with clinical variables for FVPTC patients. (**D**) Barplot documenting number of CA/IA genes that correlated with six clinical variables in FVPTC. (**E**) REVEALER plots illustrating genomic alteration associated with dysregulated genes in FVPTC. (**F**) Barplot depicting number of samples that possess the most common genomic alterations.

We discovered that in contrast to genes in CPTC, dysregulated genes in FVPTC do not cluster functionally in relation to *p53* effectors or the *AP-1* transcription factor network. Instead, the CA pathways of MAPK signaling and TNF signaling as well as the IA pathways of T-cell/B-cell signaling and Jak-STAT signaling are most prominently dysregulated in FVPTC (Figure 3A). Furthermore, the expressions of *MUC1*, *FN1*, and *S100*-family genes do not correlate with the clinical variables of FVPTC patients. Instead, the CA/IA gene *APLN* and IA gene *IL16* associated strongly with pathologic stage, pathologic T stage, and pathologic M stage (Figure 3B,C). *IL16* is a cytokine that recruits CD4^+^ immune cells, and its downregulation in FVPTC can suggest immunosuppression in the tumor microenvironment. We also found that even though less IA gene expression correlation to clinical variables can be found in FVPTC than in CPTC, more genes associated with metastasis in FVPTC, which could be a result of immunosuppression at metastasis sites by FVPTC cells (Figure 3D). 

No correlation to genomic alterations was observed for *APLN* or *IL16*, but some upregulated CA genes with no correlation to clinical variables exhibited correlations for REVEALER (Figure 3E). This may suggest that genomic alterations play a less important role in FVPTC phenotype. FVPTC samples also exhibit less commonality in genomic alterations than CPTC samples. Over 70% of FVPTC samples have the *BRAF* mutation. The most common mutations besides *BRAF* were *MUC*, *MXRA5*, and *RAS* mutations, although very few samples possess them (Figure 3F). The most common copy number variations were amplifications at the 13q14, 13q34, and 9q34 loci and deletions at the 15q25 locus. The 13q14 deletion has previously been implicated in a possible epigenetic tumor-suppressor mechanism [26], while multiple deletions at the 13q34 locus have been implicated in head and neck squamous cell carcinoma [27]. Alterations at the 15q25 locus are related to an increased risk for lung cancer development [28].

### 2.6. RAET-Family Genes and Antigen Presentation Pathways Define the TCPTC Dysregulation Landscape

**Figure 4:**
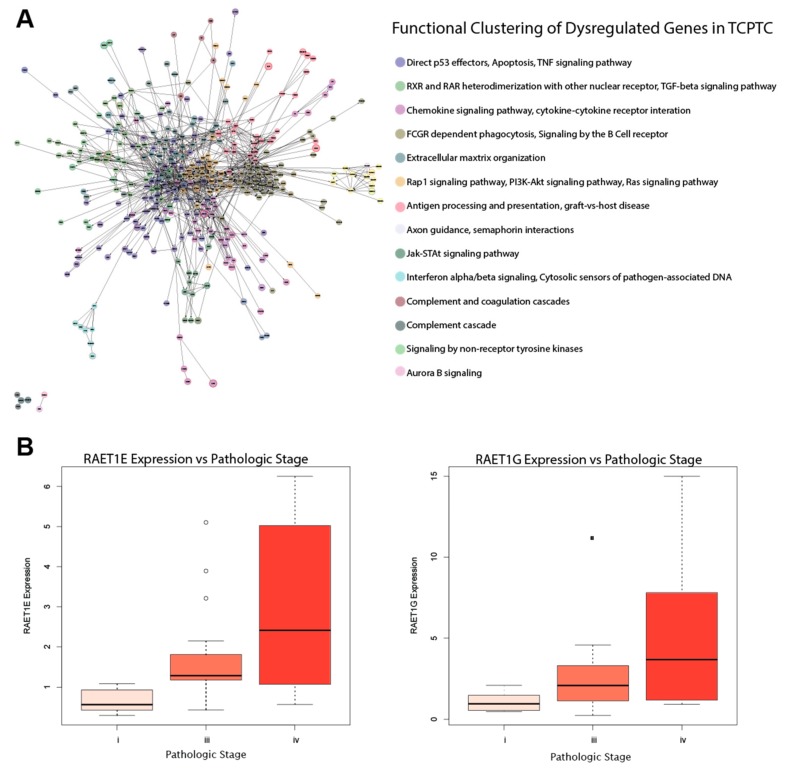
Pathways and genes characterizing the TCPTC dysregulation landscape. (**A**) ReactomeFIViz functional clustering analysis for dysregulated genes in TCPTC vs normal samples. (**B**) Boxplots presenting association of *RAET1E and RAEIG* expression with clinical variables.

We found most pathways dysregulated in TCPTC, including TNF signaling, RXR/RAR pathways, and chemokine signaling, to be similar to those found in the two other PTC subtypes (Figure 4A). However, the IA pathways of *FCGR* dependent phagocytosis and antigen processing and presentation are uniquely dysregulated in TCPTC. The *FCGR*-related genes are mostly downregulated and include key B-cell and antibody production-related genes, while antigen processing and presentation genes are mostly upregulated (Figure 4A). Because antibodies are produced against antigens, the dysregulation between the two pathways may be interrelated. 

While TCPTC exhibited the largest number of dysregulated CA and IA genes, only the expressions of *RAET1E* and *RAET1G* correlated with clinical variables—specifically pathologic stage (Figure 4B). The retinoic acid early transcript (RAET) proteins are stress-induced ligands for the immune cell activating receptor NKG2D [29]. While the RAET ligands can signal immune recognition of cancer cells, it is likely that immunosuppression within the microenvironment prevents effective RAET signaling in TCPTC, and the upregulation of NKG2D ligands have been linked to poor prognoses in other cancers [30,31].

The TCPTC group could not be used to produce conclusive REVEALER results due to the low number of patients used. Therefore, the TCPTC group was excluded from this portion of the analysis.

### 2.7. Differentially expressed genes between PTC subtypes exhibit strong immune association

**Figure 5:**
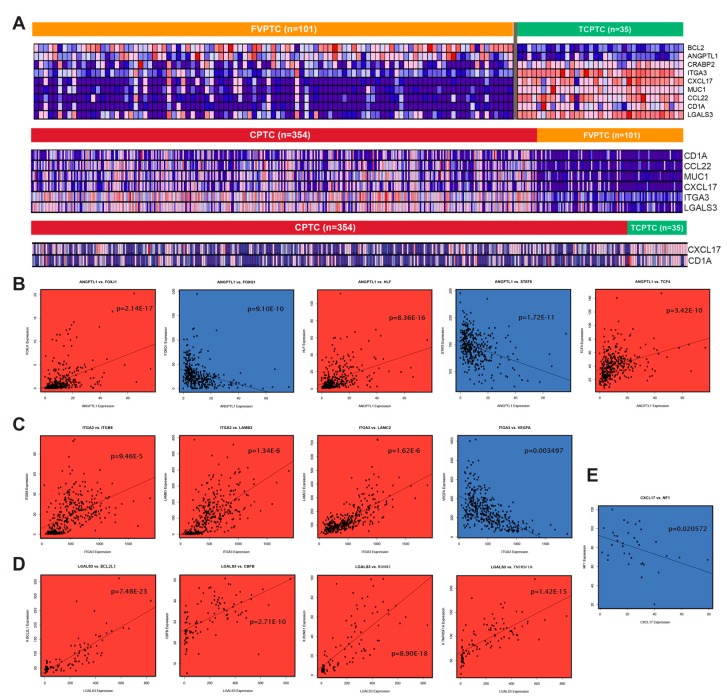
Analysis of gene expression differences between PTC subtypes. (**A**) Heatmap visualizing the differential expression of a panel of important CA/IA genes after comparing the three PTC subtypes against each other. (**B**) Scatter plot of ANGPTL1 expression vs. expression of related genes in CPTC samples. (**C**) Scatter plot of ITGA3 expression vs. expression of related genes in CPTC samples. (**D**) Scatter plot of LGALS3 expression vs. expression of related genes in FVPTC samples. (**E**) Scatter plot of CXCL17 expression vs. NF1 expression in TCPTC samples.

Besides comparing PTC samples to normal samples, we also performed differential expression analysis between the different variants of PTC and discovered a panel of genes strongly upregulated in TCPTC compared to FVPTC (Figure 5A). This gene panel consists of IA genes *CXCL17*, *CCL22*, *CD1A*, *LGALS3*, and *ITGA3*, as well as the CA genes *ITGA3* and *MUC1*. Two genes that are both IA and CA, *BCL2* and *ANGPTL1*, are more highly expressed in FVPTC than TCPTC. A comparison of CPTC to FVPTC reveals that *CD1A*, *CCL22*, *CXCL17*, *MUC1*, *LGALS3*, and *ITGA3* are more highly expressed in CPTC, but other genes differentially expressed between FVPTC and TCPTC, namely *BCL2*, *ANGPTL1*, and *CRABP2*, are not different between CPTC and FVPTC. Finally, a comparison of CPTC to TCPTC reveals that *CXCL17* and *CD1A* are both upregulated in TCPTC, but no other genes from the initial panel were significantly different in expression. Our results are consistent with the findings above, where it was found that the dysregulation landscape of TCPTC is most different from that of FVPTC and relatively similar to that of CPTC.

We plotted the expressions of these genes against the expressions of genes within the same pathways and presented the highest correlations (Figure 5B–E). We found that the genes exhibited strong correlations with other CA and IA genes. *ANGPTL1* expression is associated with the expressions of CA genes *HLF*, *TCF4*, *FOXJ1*, and *FOXQ1* as well as expression of IA gene *STAT6* in CPTC (Figure 5B). Interestingly, the expression of *ITGA3* is associated with downregulation of the oncogene *VEGFA* in CTPC, suggesting that the VEGF pathway may not be involved in conferring the aggressive phenotypes of CTPC and TCPTC (Figure 5C). VEGF pathways are also not enriched by functional gene clustering (Figure 2A). In FVPTC samples, *LGALS3*, an IA gene, is strongly associated with genes associated with blood cancers (*RUNX1*, *BCL2L1*, *CBFB*, and *TNFRSF1A*), suggesting a strong immune association in LGALS3 activity (Figure 5D). Finally, in TCPTC samples, *CXCL17* expression is inversely associated with that of *NF1*, a gene that downregulates the *RAS* oncogene (Figure 5E).

### 2.8. miRNA-mediated silencing of FN1 in CPTC and ITGA3 in FVPTC

Gene set enrichment analysis (GSEA) was used to identify microRNAs (miRNAs) that silence IA genes by identifying when upregulation of miRNAs corresponded with downregulation of CA and IA genes.

**Figure 6:**
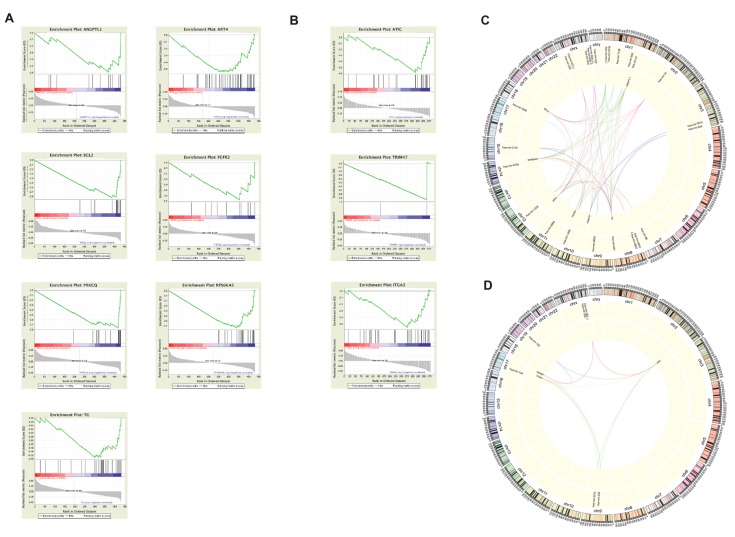
miRNA silencing of dysregulated CA/IA genes. GSEA analysis for (**A**) the CPTC vs normal comparison and (**B**) the FVPTC vs normal comparison. miRNA silencing is indicated by a downward-facing peak at the left extreme of the plot. (**C**,**D**) Circos plots for CPTC and FVPTC, respectively, visualize connections between multi-target miRNAs (more than one target) and the genes that they potentially silence.

For the CPTC subtype analyses, seven genes (ANGPTL1, ART4, BCL2, FGFR2, PRKCQ, RPS6KA5, and TG) yielded plots that suggested potential miRNA silencing (Figure 6A). Notably, the differences in dysregulation of *TG* between subtypes coincide with patterns revealed by GSEA. In previous studies, a connection has been established between the expression of *TG* and *FN1*, in which trans-acting or protein-protein interactions affect the development of thyroid cancer [32]. Differential expression analysis found that *FN1* was upregulated more than 10-fold in CPTC but was upregulated only six-fold in FVPTC when compared to normal samples. The CPTC vs FVPTC differential expression analysis validates this difference, with *FN1* having logFC = 2.838 (Table S2). Additionally, *FN1* is commonly upregulated in aggressive forms of thyroid cancer [18]. Combining this information suggests a mechanism involving both *TG* and *FN1*. Specifically, *TG* may regulate the expression of *FN1*, but is unable to do so when acted upon by miRNAs. Therefore, the upregulation of *FN1* via *TG* silencing may be indicative of a disease pathway unique to CPTC.

For the FVPTC subtype, GSEA for *ATIC*, *ITGA3*, and *TRIM47* suggested potential gene silencing by miRNAs (Figure 6B). *ITGA3*, a member of the integrin alpha chain family of proteins that is normally highly expressed in thyroid tissue, is downregulated in FVPTC in comparison to its expression in other subtypes. miRNA silencing may be key to the suppression of *ITGA* in FVPTC. miRNAs have been shown to silence *ITGA3* expression, and in turn inhibit cancer development, in head and neck squamous cell carcinoma [33]. 

For the TCPTC subtypes, there were no GSEA plots that yielded the characteristic trend that indicates the desired correlation of low gene expression with high miRNA expression. Based on these results, miRNAs seemingly do not have a significant effect on gene expression in TCPTC patients. 

miRNAs that likely target more than one dysregulated genes were visualized. We found that a large number of multi-target miRNAs are present in CPTC (Figure 6C), while much fewer are present in TCPTC (Figure 6D). Interestingly, many multi-target miRNAs are located in chromosome X.

### 2.9. Validation of RNA-sequencing data supports CPTC and FVPTC dysregulation patterns

**Figure 7:**
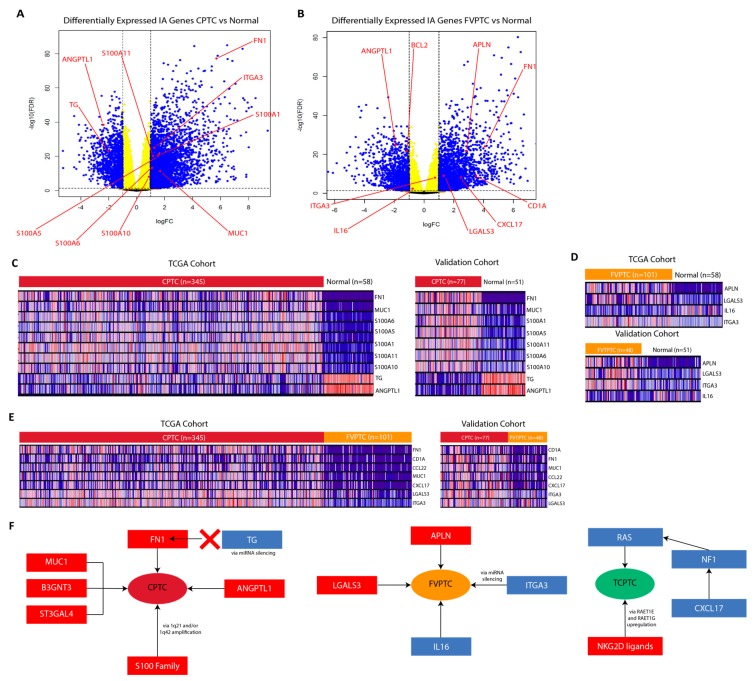
Validation of CPTC and FVPTC CA and IA gene dysregulation. Volcano plots for (**A**) the CPTC vs normal comparison and (**B**) the FVPTC vs normal comparison using the validation RNA-seq data. Genes of interest are indicated with red labels. Heatmaps comparing the landscapes of significantly dysregulated CA and IA genes between the validation cohort and TCGA cohort for (**C**) the CPTC vs normal comparison, (**D**) the FVPTC vs normal comparison, and (**E**) the CPTC vs. FVPTC. (**F**) Summary of most important findings. Arrows indicate possible interactions suggested by analysis and previous studies, red boxes indicate upregulation, and blue boxes represent downregulation.

We utilized sequencing data from Yoo et al. to validate the gene expression dysregulation we found in CPTC and FVPTC through TCGA data [34]. We were not able to include TCPTC sequencing data in the validation step due to lack of another sequencing dataset. From analysis of the validation cohorts, we found that IA and CA genes identified in our previous analyses to be dysregulated in CPTC and FVPTC subtypes were almost all significantly dysregulated in the same direction in the validation cohorts (Figure 7A–D). For a few genes—specifically ITGA3, IL16, and S100A10—the dysregulation is significant statistically, but the fold change did not pass the threshold of significance (<−2 or >2 folds, Figure 7A,B). In addition to comparing cancer samples against normal samples, we have also compared CPTC samples to FVPTC samples and obtained similar dysregulation trends for clinically relevant IA and CA genes in both the TCGA and the validation patient cohorts (Figure 7E).

We summarize the most significant and clinically relevant dysregulations we uncovered in each subtype of PTC in Figure 7F.

## 3. Discussion

PTC is the only cancer with an increase in incidence for all ethnicities. Furthermore, there is an increased rate of diagnosis at late stages of PTC development. Although significant progress has been made towards characterizing the landscape of PTC, there is little information distinguishing pathogenesis of different PTC subtypes. Additionally, little success has been achieved in treating patients who have more aggressive forms of PTC such as TCPTC, especially if patient tumors lack the *BRAF* mutation [19]. Thus, it is imperative that key distinguishing features be identified for PTC subtypes in order to elucidate mechanisms that cause altered prognosis and that may serve as targets for immunotherapy.

We profiled CA and IA pathways that are similar or different in PTC subtypes on the scales of individual genes, functional gene clusters, genes associated with genomic alterations, and genes associated with miRNA regulation. Through this multi-scale data, we propose that while CA and IA dysregulations are present in all subtypes, the CA pathways may be main drivers of CPTC’s phenotype, while IA pathways may be main divers of TCPTC’s phenotype. FVPTC may be driven by both CA and IA dysregulations. In CPTC, CA genes (*FN1*, *S100*-family members, and *MUC1*) account for most of the genes with both clinical variable associations and correlation with genomic alteration events, which are drivers of carcinogenesis. Furthermore, the largest functional pathways dysregulated are cancer-related in CPTC. In contrast, large fractions of dysregulated genes are part of IA functional pathways in TCPTC and FVPTC. The upregulation of several IA genes differentiates TCPTC from the other subtypes, and only the dysregulation of *RAET*-family genes, central to immune-mediated cell killing, correlated with clinical prognosis in TCPTC. Lastly, a large fraction of dysregulated genes in TCPTC are related to antigen processing and antibody production, according to functional annotations. 

Commonly altered loci were identified using the REVEALER algorithm for each subtype. In general, the outputs indicated that CPTC was commonly implicated with alterations at the 1q21, 1q42, 5p15, and 9q34 loci. FVPTC also had increased CNVs at the 9q34 loci, as well as at the 13q14 and 13q34 loci. These loci have been implicated in the progression of other metastatic cancers. Specifically, the 1q21 locus has been correlated with cancer progression along with *MUC1* upregulation [21], further contributing to the proposed role *MUC1* has in aggressive PTC phenotype. The *BRAF* mutation, as well as alterations at the 1q42 and 1q21 loci, corresponded with the dysregulation of *S100 genes* in CPTC. Additionally, *FN1* upregulation corresponded with high *BRAF* and 1q21 alteration frequency. These relationships build on the potential significance of these genes in CPTC pathogenesis. 

Silencing of IA gene expression mediated by microRNAs was identified using GSEA. In the CPTC group, *TG* was identified as a promising target of miRNA silencing, with such activity correlating more to the downregulation of TG in CPTC in comparison to the other PTC subtypes. *TG* silencing could also be related to the corresponding dysregulation of *FN1*. Specifically, downregulation of *TG* via miRNAs coincides with a comparatively lower expression of *FN1*, which is commonly upregulated in aggressive forms of thyroid cancer. This may indicate a transcriptomic feature that causes CPTC to be less aggressive than TCPTC. Additionally, *MUC1*, which is involved in TGF-beta signaling from ReactomeFiViz annotations, may also be implicated in this pathway, as *FN1* is inducible by TGF-beta [12]. The downregulation of *MUC1* in CPTC compared to TCPTC and subsequent potential inactivity of TGF-beta receptor pathways may induce the altered regulation of *FN1* and *TG*. Considering that *MUC1* upregulation and *BRAF* mutation presence correlate in thyroid cancer [17], our proposed mechanism provides a pathway for the *BRAF* mutation to drive PTC pathogenesis and progression.

In conclusion, each subtype of PTC possesses a unique dysregulation landscape implicating CA and IA pathways, with CA pathways being more dysregulated in CPTC and IA pathways being more dysregulated in TCPTC, relative to each other. Experiments controlling for CA or IA pathways’ dysregulation in each subtype will be needed to elucidate the importance of CA and IA pathways in contributing to the cancer’s phenotype, in order to inform whether tumor-intrinsic pathways or immune pathways should be targeted for prevention of metastasis and elimination of aggressive phenotypes.

## 4. Materials and Methods 

### 4.1. The Cancer Genome Atlas (TCGA) RNA-sequencing datasets and cohort designation

Level 3-normalized mRNA expression read counts for tumor samples from 345 CPTC patients, 101 FVPTC patients, and 35 TCPTC patients along with the patients’ clinical data were downloaded from TCGA [35] on 2 February, 2018. To create a normal group to compare to the cancer groups, mRNA read counts for adjacent solid tissue samples of 58 PTC patients were also downloaded. Patients from TCGA study were followed until their time of death or for the maximum duration of the study—which was 4684 days. Data on patient disease progression, drug administration, and vital status recorded over this period was used in later analysis. Patient cohorts were created from downloaded data in order to compare PTC subtypes to each other and to normal tissue samples. These cohorts consisted of CPTC vs Normal, FVPTC vs Normal, TCPTC vs Normal, CPTC vs FVPTC, CPTC vs TCPTC, and FVPTC vs TCPTC. The former three cohorts were used to identify genomic and transcriptomic differences between PTC subtypes and normal controls while the latter three cohorts were used to find differences in gene expression levels and clinical variable correlation between PTC subtypes. Volcano plots were created using edgeR and inputting FDR and logFC values from the differential analysis.

### 4.2. Differential expression analysis to identify dysregulated immune-associated genes

Using edgeR v3.5 [36], mRNA read count inputs were filtered, resulting in the removal of lowly expressed mRNAs (counts-per-million <1 when comparing samples from the larger group to those of the smaller group in a cohort) from the analysis. Trimmed mean of M-values (TMM) were normalized and pairs of mRNAs were designated to identify those that were significantly differentially expressed when comparing one cohort to another. mRNAs considered to be significantly dysregulated were those with fold change >2 or <−2 and false discovery rate (FDR) <0.05, output by the edgeR analysis. After filtering for dysregulated genes, potential candidates were retained if they were considered to be immune associated. Genes are determined to have immune association if they are involved in adaptive or innate immune processes or in tumor antigen production. We created a list of all immune associated genes from ImmPort [37], which lists all genes involved in adaptive or innate immune processes, the InnateDB [38], which lists genes associated with innate immunity, and the TANTIGEN database [39], which list CA genes.

### 4.3. Functional Clustering Via ReactomeFIViz

The Cytoscape software was used in tandem with the ReactomeFIViz plugin to create clusters of gene nodes, implying similarity of function between nodes of similar modules. Fold change data was used to adjust node details such as size and outline. Linked genes, as depicted in Figure 1D, were taken from the program’s database by inputting a list of the IA genes of interest. Significant functions of modules given by the clustering software were determined by FDR <0.05.

### 4.4. Comparing gene expression to clinical variables

Clinical significance of immune-associated genes was determined by employing the Kruskal-Wallis test. Gene expression values were correlated with variables including pathologic stage, pathologic TMN stage, residual tumor, histologic type, lymphocyte infiltration, monocyte infiltration, and neutrophil invasion. In analyzing the pathologic TMN stages as individual variables, stages such as T1a and T1b were grouped together as stage T1. Because mortality rates of THCA are generally low, patients’ MAICS scores were used to correlate immune-associated gene expression with prognosis with patients being assigned to one of four bins designated by their scores, with higher scores corresponding with lower survival expectancy and advanced prognosis.

### 4.5. Correlating gene expression with genomic alterations

CNV and mutation data was obtained from annotation files generated by the BROAD Institute GDAC Firehose on March 31, 2018. The surface-level trends of mutation presence were analyzed by calculating the percentage of patients with each mutation, indicated by a binary value per mutation. The GDAC files were compiled into input files for the REVEALER (repeated evaluation of variables conditional entropy and redundancy) algorithm, which identifies sets of specific CNVs and mutations that are most likely implicated in changes to the target expression profile. The target profile was identified as the expression of a single immune-associated gene. The REVEALER algorithm runs in multiple iterations in order to identify the most prominent genomic alterations. For our study, we set the maximum number of iterations to three. The algorithm also allows for the use of a seed, or a particular mutation of CNV gain or loss event that may account for target activity. However, because we did not know the individual genetic alterations that were responsible for each IA genes’ dysregulation, the seed was set to null. Significant association between genomic alteration and gene expression was determined by conditional information coefficient (CIC) >0.25 and *p*-value < 0.05.

### 4.6. Association of gene expression with microRNA silencing

To determine possible silencing of immune-associated gene by microRNAs, a list of differentially expressed microRNAs was created in a similar fashion to the compilation of dysregulated genes described previously, instead using miRNA read count files from TCGA. Input files for the Gene Set Enrichment Analysis (GSEA) were created, detailing the miRNAs that act on each IA gene, miRNA read counts, and mRNA read counts. The miRNAs that act on each gene were determined by consulting the TargetScan database [40], which predicts miRNA activity based on conserved sites on DNA and corresponding miRNA. The GSEA program determines whether the miRNAs that act on a single gene show significant differences in expression when coupled with dysregulation of the gene. The program assigns enrichment scores to each miRNA based on the expression of the target gene, assigning high enrichment scores when high miRNA expression correlated with high gene expression. In addition, the miRNAs are ranked based on their level of dysregulation, resulting in a final list that ranks miRNAs from highest to lowest dysregulation. A final enrichment plot is created that compares miRNAs’ list ranks to their enrichment scores. Because miRNAs silence gene expression, high list ranks must correlate to low enrichment scores in order for a significant relationship to be identified. miRNAs are determined to influence gene expression if the nominal *p*-value for a negative correlation between list rank and enrichment score is <0.05. Circos plots were generated from GSEA results to link analyzed miRNAs to the genes that they may regulate. The plots were created by inputting miRNA and IA gene genome location data into the Circos software [41].

### 4.7. Determination of co-Expression between dysregulated genes and related genes

To determine the effect of IA or CA gene dysregulation on other genes that share common pathways with the CA/IA genes, lists of genes in the same pathways as selected CA/IA genes were obtained from the Pathway Commons [42]. Lists were filtered for interactions in which CA/IA genes acted on or were acted on by other genes. CPM data for the genes from the remaining relationships was compared to that of the CA/IA genes using edgeR. Possible co-expression was indicated by plots with *p*-value < 0.05.

### 4.8. Validation of TCGA RNA-sequencing datasets

Using RNA-sequencing data performed on CPTC, FVPTC and adjacent normal thyroid tissue in Yoo et al. [34], differential expression analysis, as described above, was completed in order to validate results obtained using TCGA datasets. Datasets for 77 CPTC samples, 48 FVPTC samples, and 81 matched adjacent normal samples were available. The STAR RNA-seq aligner [43] was used to convert the RNA-sequencing data into a counts format.

## 5. Conclusions

We conclude that several immune and cancer-associated genes and pathways define an RNA-expression dysregulation landscape that is unique to each individual PTC variant. Specifically, *MUC1*, *S100*-family genes, *FN1*, and several CA pathways characterize CTPC; *APLN*, *IL16*, MAPK signaling, and several IA pathways characterize FVTPC; and RAET-family genes and the antigen presentation pathway characterize TCPTC. We also implicate miRNAs in the downregulation of certain genes and present a possible mechanism for miRNA-mediated suppression of *TG*.

## Supplementary Materials

The following are available online at https://www.mdpi.com/2072-6694/11/8/1179/s1, Table S1: classical vs normal differentially-expressed IA and CA genes, Table S2: follicular vs normal differentially-expressed IA and CA genes, Table S3: normal vs tall cell differentially-expressed IA and CA genes, Table S4: classical vs follicular-variant differentially-expressed IA and CA genes, Table S5: classical vs tall cell differentially-expressed IA and CA genes, Table S6: follicular vs tall cell differentially-expressed IA and CA genes, Figure S1: non-invasive FVPTC vs invasive FVPTC differentially-expressed IA genes.
