# Supplementary figures and images for: Papillary Thyroid Carcinoma Variants are Characterized by Co-dysregulation of Immune and Cancer Associated Genes
Source: Cancers (Basel). 2019 Aug 15;11(8):1179. doi: 10.3390/cancers11081179 (PMC6721495; doi:10.3390/cancers11081179)

A

Differentially Expressed IA Genes niFVPTC vs iFVPTC

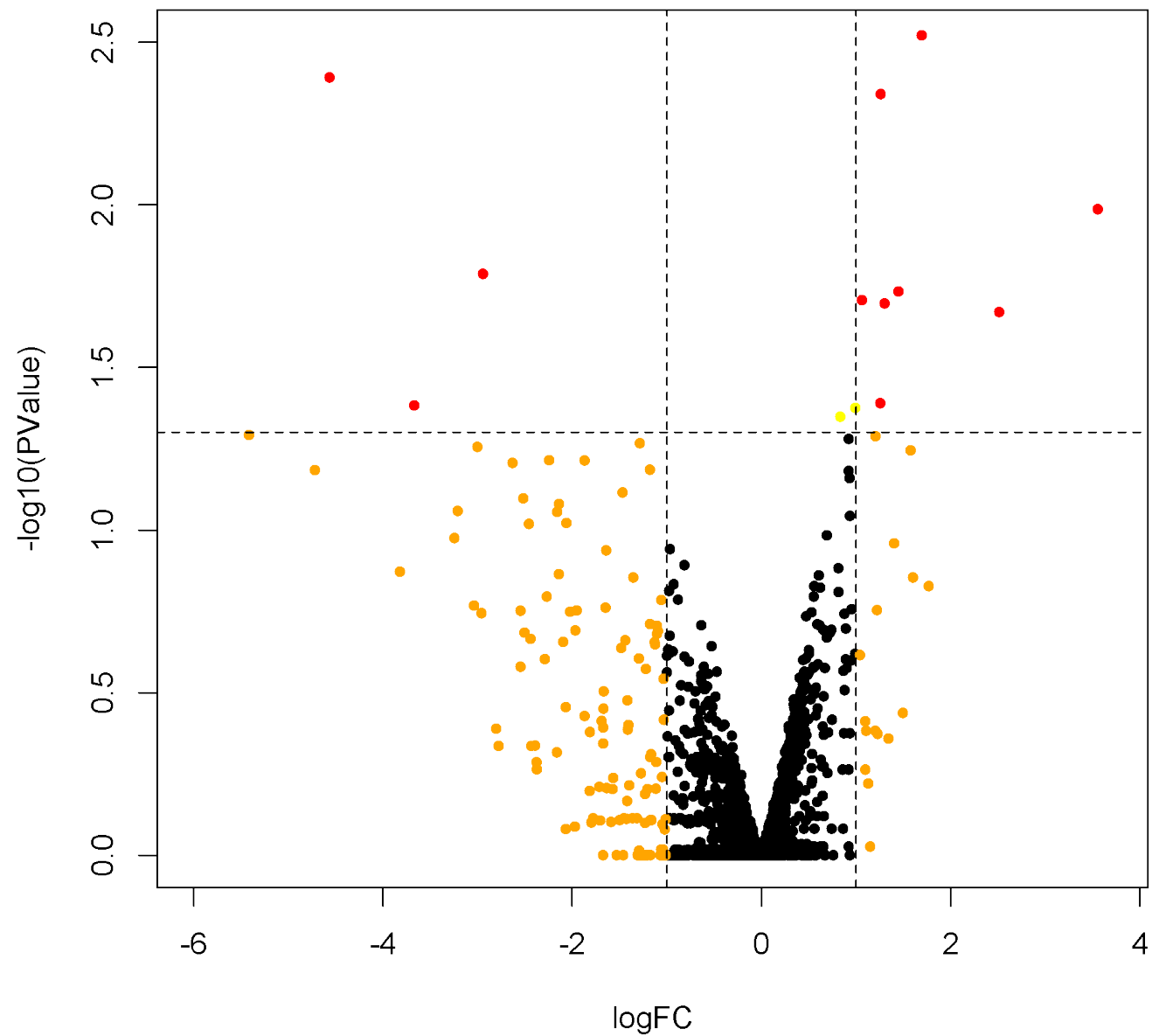

B

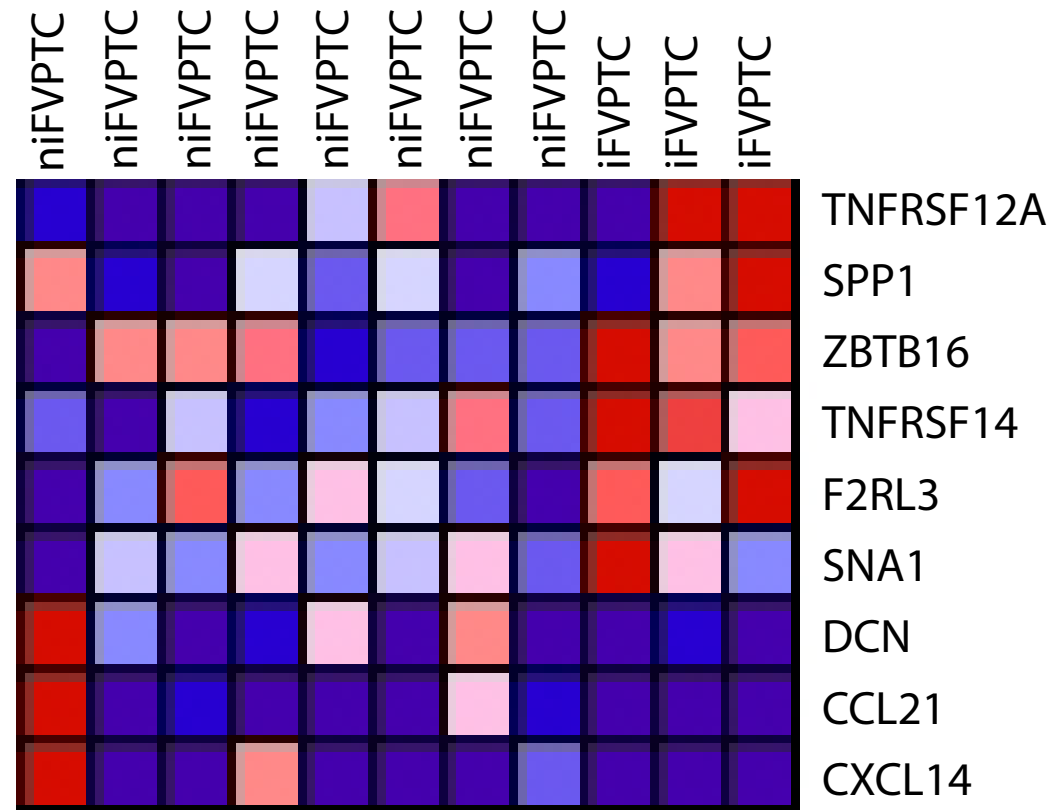

Supplement: Supplementary file 1 [file cancers-11-01179-s001.zip › cancers-554386-supplementary/cancers-554386-suppply-final/Figure S1.pdf]
